# Expression and Activation by Epstein Barr Virus of Human Endogenous Retroviruses-W in Blood Cells and Astrocytes: Inference for Multiple Sclerosis

**DOI:** 10.1371/journal.pone.0044991

**Published:** 2012-09-27

**Authors:** Giuseppe Mameli, Luciana Poddighe, Alessandra Mei, Elena Uleri, Stefano Sotgiu, Caterina Serra, Roberto Manetti, Antonina Dolei

**Affiliations:** 1 Department of Biomedical Sciences and Centre of Excellence for Biotechnology Development and Biodiversity Research, University of Sassari, Sassari, Italy; 2 Department of Neurosciences and MIS, University of Sassari, Sassari, Italy; 3 Department of Clinical, Experimental and Oncological Medicine, University of Sassari, Sassari, Italy; Institute Biomedical Research August Pi Sunyer (IDIBAPS) – Hospital Clinic of Barcelona, Spain

## Abstract

**Background:**

Proposed co-factors triggering the pathogenesis of multiple sclerosis (MS) are the Epstein Barr virus (EBV), and the potentially neuropathogenic MSRV (MS-associated retrovirus) and syncytin-1, of the W family of human endogenous retroviruses.

**Methodology/Principal Findings:**

In search of links, the expression of HERV-W/MSRV/syncytin-1, with/without exposure to EBV or to EBV glycoprotein350 (EBVgp350), was studied on peripheral blood mononuclear cells (PBMC) from healthy volunteers and MS patients, and on astrocytes, by discriminatory *env*-specific RT-PCR assays, and by flow cytometry. Basal expression of HERV-W/MSRV/syncytin-1 occurs in astrocytes and in monocytes, NK, and B, but not in T cells. This uneven expression is amplified in untreated MS patients, and dramatically reduced during therapy. In astrocytes, EBVgp350 stimulates the expression of HERV-W/MSRV/syncytin-1, with requirement of the NF-κB pathway. In EBVgp350-treated PBMC, MSRV*env* and syncytin-1 transcription is activated in B cells and monocytes, but not in T cells, nor in the highly expressing NK cells. The latter cells, but not the T cells, are activated by proinflammatory cytokines.

**Conclusions/Significance:**

*In vitro* EBV activates the potentially immunopathogenic and neuropathogenic HERV-W/MSRV/syncytin-1, in cells deriving from blood and brain. *In vivo*, pathogenic outcomes would depend on abnormal situations, as in late EBV primary infection, that is often symptomatic, or/and in the presence of particular host genetic backgrounds. In the blood, HERV-Wenv activation might induce immunopathogenic phenomena linked to its superantigenic properties. In the brain, toxic mechanisms against oligodendrocytes could be established, inducing inflammation, demyelination and axonal damage. Local stimulation by proinflammatory cytokines and other factors might activate further HERV-Ws, contributing to the neuropathogenity. In MS pathogenesis, a possible model could include EBV as initial trigger of future MS, years later, and HERV-W/MSRV/syncytin-1 as actual contributor to MS pathogenicity, in striking parallelism with disease behaviour.

## Introduction

Multiple sclerosis (MS) is a chronic neurological disease, which usually begins in early adulthood. It causes repeated unpredictable bouts of motor disorders, partial paralysis, sensory abnormalities and visual impairment, with demyelination and gliosis, various degrees of axonal pathology and episodic or progressive neurological disability. The aetiology is unknown and complex, but results from an inflammatory process that, among other effects, attacks and destroys oligodendrocytes, the cells that form the myelin sheaths around axons in the brain and spinal cord [1]. The immunopathogenic phenomena leading to MS are thought to be triggered by an environmental (viral?) factor operating on a predisposing genetic background.

The most consistent studies for a potential virus involvement in MS exist for the Epstein Barr virus (EBV) [2]–[3], and for two members of the W family of human endogenous retroviruses (HERV-W), reviewed in [4]–[7]. The MSRV element (MS-associated retrovirus) is the first known member of the HERV-W family [8–]; it has been detected and purified from cells of MS patients, as free virus-like particles, carrying RT activity and an RNA genome with terminal repeats, *gag*, *pol* and *env* regions. The other HERV-W member related to MS is, a replication-incompetent element located on chromosome 7q21–22, that has inactivating mutations in the gag and pol genes and is not able to form virus like particles. The env product of ERVW-1 has been named syncytin-1, since it is produced by placental trophoblasts and causes their fusion to form the syncytial layer, during pregnancy [9]. The syncytin-1 protein can be found intracellularly and on the plasma membrane, but has not been detected extracellularly, nor its RNA sequence in virus-like particles.

MSRVenv and syncytin-1 proteins share several biological features, and are potentially pathogenic: they have pro-inflammatory and superantigenic properties, and have been shown to cause neurotoxic effects *in vitro* and in humanized or transgenic animal models [10]–[11]: they may cause neuroinflammation, neurodegeneration, alterations of the immune system and stress responses; both have been suggested as co-factors triggering the immuno-pathogenesis of MS. Expression of HERV-W/MSRV/syncytin-1 occurs in astrocytes of MS lesions of the brain [11]–[12], as well as in endothelial and microglial cells [13]. In a mouse model, oligodendrocytes (which produce the myelin sheath of the nerve) were shown to be sensitive to syncytin-mediated release of redox reactants from astrocytes [11].

Studies from our group found repeatedly retrovirus-like MSRV particles and MSRV-specific mRNA sequences in MS patients, and MRSV presence/load strikingly paralleled disease behaviour. A large multicentre study of MS patients and controls from different European areas showed that MSRV presence and load in blood and spinal fluid was significantly associated to MS in all ethnic groups [14]. We have found direct parallelisms between MSRV positivity/load (in blood, spinal fluid, and brain samples) and MS temporal and clinical stages, as well as active/remission phases: MSRV positivity of spinal fluids increased with MS duration [15] and its presence in early MS was related to worse prognosis in the next ten years: starting from comparable conditions, after three [16], six [17], and ten years [18], mean disability, annual relapse rate, therapy requirement and progression to secondary-progressive MS were significantly higher in patients starting with MSRV-positive spinal fluids. A longitudinal evaluation of patients, during efficacious therapy with interferon (IFN)*β*, revealed that viremia fell rapidly below detection limits; notably, a patient, after initial clinical and virological benefit, had MSRV rescue, *preceding* strong disease progression and therapy failure [19]; it was proposed that evaluation of plasmatic MSRV could be considered the first prognostic marker for the individual patient, to monitor disease progression and therapy outcome. This possibility is reinforced by the study of patients with optic neuritis, a disease frequently prodromic to MS: MSRV positivity of patients was found significantly higher than that of pathological controls, and the conversion to full-blown MS in the next 20 months occurred only among MSRV-positive patients [20].

In line with our findings, an independent 1-year follow up study of MS patients observed significant decreases in anti-HERV-Wenv and anti-HERV-Henv antibody reactivity as a consequence of IFN-β therapy, closely linked to efficacy of therapy/low disease activity [21].

As for EBV, the risk of MS is low in EBV-negative individuals, and increases several folds following EBV infection, particularly if the EBV infection occurs in late adolescence or adulthood, when the infection is symptomatic. In facts, in 35 to 50 per cent of the cases, this delayed EBV primary infection causes infectious mononucleosis (IM), that has been associated with a two-to-four fold increased risk of MS, [22]–[26]. There is also an increased risk of MS among EBV-positive children [27]. A meta-analysis showed an association between the appearance of anti-EBV antibodies and the MS onset, 5–20 years later [2]; another one reported that the relative risk of MS for a past history of IM is 2.17 [3].

There is convincing epidemiological evidence that late EBV primary infection/seroconversion is a risk factor for MS development, but the relationship between EBV and MS pathogenesis remains elusive. Studies that detected EBV DNA in the brain failed to prove the association between EBV and MS [2], [28]–[31], without consistent evidence of increased copies of the EBV genome in the blood or serological evidence of reactivation [25]. Furthermore, it remains to be determined whether EBV would continue to play a role after disease initiation [22], [25].

Given the above findings, a link between EBV and HERV-W/MSRV/syncytin-1 seemed possible. Therefore we performed *in vitro* experiments on cells transcribing MSRV and syncytin-1, and with HERV-Wenv protein on the plasmamembrane, i.e. peripheral blood mononuclear cells (PBMC) from MS patients and MSRV-positive healthy donors (HD) and astrocytes, that were exposed to EBV or to its major envelope glycoprotein 350 (EBV gp350).

## Results

### *In silico* analysis of HERV-W sequences in human DNA and RNA

**Figure 1:**
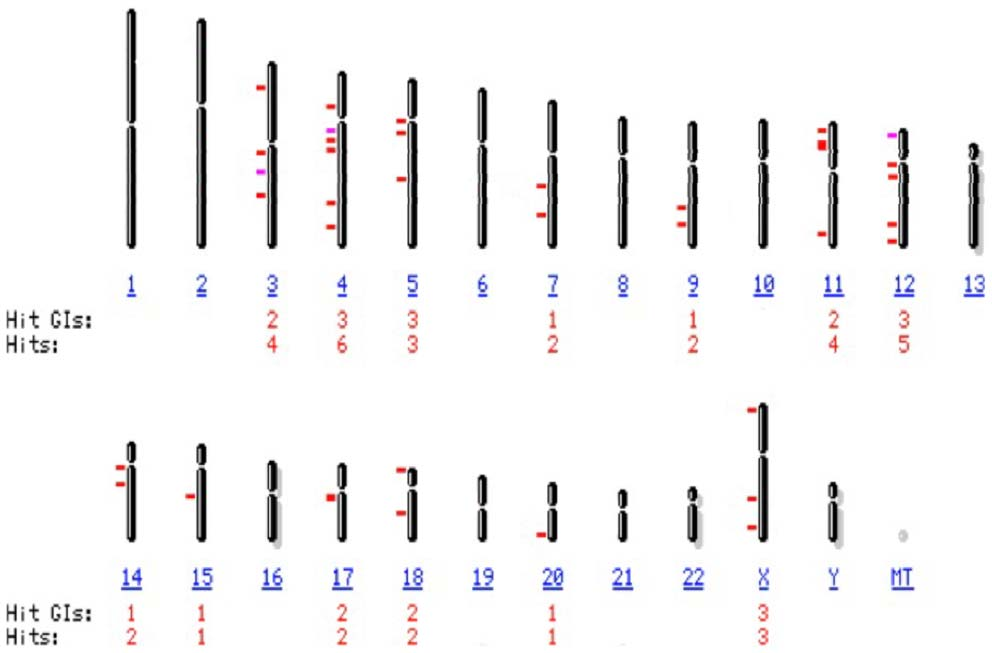
Chromosomal representation of multiple sites of integration in human DNA of HERV-W*env* loci covering ≥80% of the HERV-Wenv gene. The *in silico* analysis of the current version of the human genome was performed by NCBI Basic Local Alignment Search Tool (BLAST) program, using as queries the MSRV*env* and syncytin-1 sequences (PV14 MSRV clone, GenBank accession number AF331500, and ERVW-1*env* coding sequence NM_014590.3, respectively). From the initial BLAST-identified regions, only the sequences covering ≥80% of the HERV-Wenv gene were selected as target and are reported. Red dashes: target:score ≥200; mauves dashes: target:score 80–200. Hits: number of loci; Hit GIs: Locus number all matches; MT: mitochondrial chromosome.

Due to their repetitive nature, very few HERV elements have been characterized. As other HERV families, the W family is present in the human haploid genome in multiple copies, the majority of them highly defective, but complete proviruses have been also described, for a proposed total of ∼140–250 copies with internal sequences (more or less complete proviruses and pseudogenes with stop-codons, deletions and truncations), plus additional ∼150–340 solitary HERV-W LTRs [4], [32]–[33]. All the known HERV-W sequences are closely related, and difficult to distinguish each other [34]–[35]. For an updating of the HERV-W*env* genes that have been detected, we performed an *in silico* nucleotide BLAST search [36] in the current version of the human genome, using as queries the MSRV*env* and syncytin-1 sequences (PV14 MSRV clone, GenBank accession number AF331500, and ERVEW1*env* coding sequence NM_014590.3, respectively). From the initial BLAST-identified regions, only the sequences covering ≥80% of the genes were selected. As reported on Figure 1, HERV-W*env* elements containing ≥80% of the *env* gene are present on fourteen human chromosomes; full length HERV-W*env* sequences are present on ten chromosomes (X, 3, 4, 5, 7, 12, 14, 15, 17, 18); on Table S1a and S1b of the Supporting Information file more detailed data are reported.

At the RNA level, MSRV*env* and syncytin-1 share >94% identities [12]; at the protein level, no antibody specific for a unique HERV-W has been identified so far [5]. The homology between MSRV*env* and syncytin-1 originated debates in the attribution of possible pathogenic effects. A major difference is that only MSRV has been detected as an extracellular virus, visualized by electron microscopy, sedimenting at retrovirus buoyant density, with RT activity and a polyA(+) RNA containing terminal repeats, *gag*, *pol* and *env* sequences, while the syncytin-1 protein has been found only intracellularly or on the plasma membrane [5]. Since PCR and RT-PCR data from the literature have been obtained by using primers unable to discriminate HERV-Wenv elements, we developed discriminatory real-rime PCR assays, that could selectively amplify either MSRV*env* or syncytin-1 sequences. Reliability of the assays with respect to specificity, sensitivity and inter- and intra-assay variations were published [34].

On Table 1 an *in silico* analysis of the human DNA sequences recognized by the above MSRVenv- and syncytin-1 specific primers, and of their transcription/translation products are reported. As shown in the upper part of Table 1, apart from the virionic MSRVenv RNA sequences, the BLAST analysis showed that the 166 bp fragment amplified with MSRV*env*-specific primers is present only in two full length HERV-W*env* genes, located on chromosomes Xq22.3 and 5q11.2, and in two largely defective env genes on chromosomes 8p22.1 and 10q23 (13% and 10% of the whole *env* gene, respectively). The latter two elements are not transcribed, while the former two do. The element on chromosome 5q11.2 cannot be translated, due to the presence of multiple stop codons, while the one on chromosome Xq22.3, due to a stop codon on position 115 originates two products, of 38 and 475 amino acids, respectively, instead of the 541 amino acids of the complete env protein. The syncytin-1-specific primers, as shown in the lower part of the Table 1, recognize a 100 bp amplicon present on the chromosome 7q21.22 element only, that has retained an uninterrupted open reading frame, and therefore is fully transcribed and translated in the complete protein of 537 amino acids.

**Table 1:** Env genes integrated in the human genome that are recognized by MSRV*env*- and syncytin-1-specific real-time PCR assays.

Localization HuDNA virionic RNA	Gene length§	mRNA	Start codons (position)	Stop codons (position)	Proteic products*	Comments
MSRV*env* primers:^a^ Unknown MSRV*env* (AF331500.1)°	1623	Yes	1 (1)	no	541	
Chromosome Xq22.3 (NT_011786.16)	1623	Yes	2 (1, 196)	1 (117)	38, 475	[88]
Chromosome 5q11.2. (NT_006576.16)	1631	Yes	no	30	no	[70]
Chromosome 8p22.1 (NW_001839126.2)	212	no	no	2	no	
Chromosome 10q23 (NT_030059.13)	168	no	no	2	no	
Syncytin-1 primers: ^b^ Chromosome 7q21–2 (NM_014590.3)	1611	Yes	1 (1)	no	537	ERVW-1*env*

a BLAST program, using as query the MSRV*env* amplicon; * amino acids; § nucleotides; ° Accession number; ^b^ BLAST program, using as query the syncytin-1 amplicon.

### Basal expression of HERV-Wenv/MSRVenv/syncytin-1 by astrocytes and blood cells *in vitro* and *in vivo*

Previously we have shown HERV-Wenv protein immuno-staining in up to 50% of the astrocytes of active lesions in the brain of MS patients [12]. Therefore U87-MG glioblastoma-astrocytoma cells were analysed for transcription and translation of HERV-W elements.

**Figure 2:**
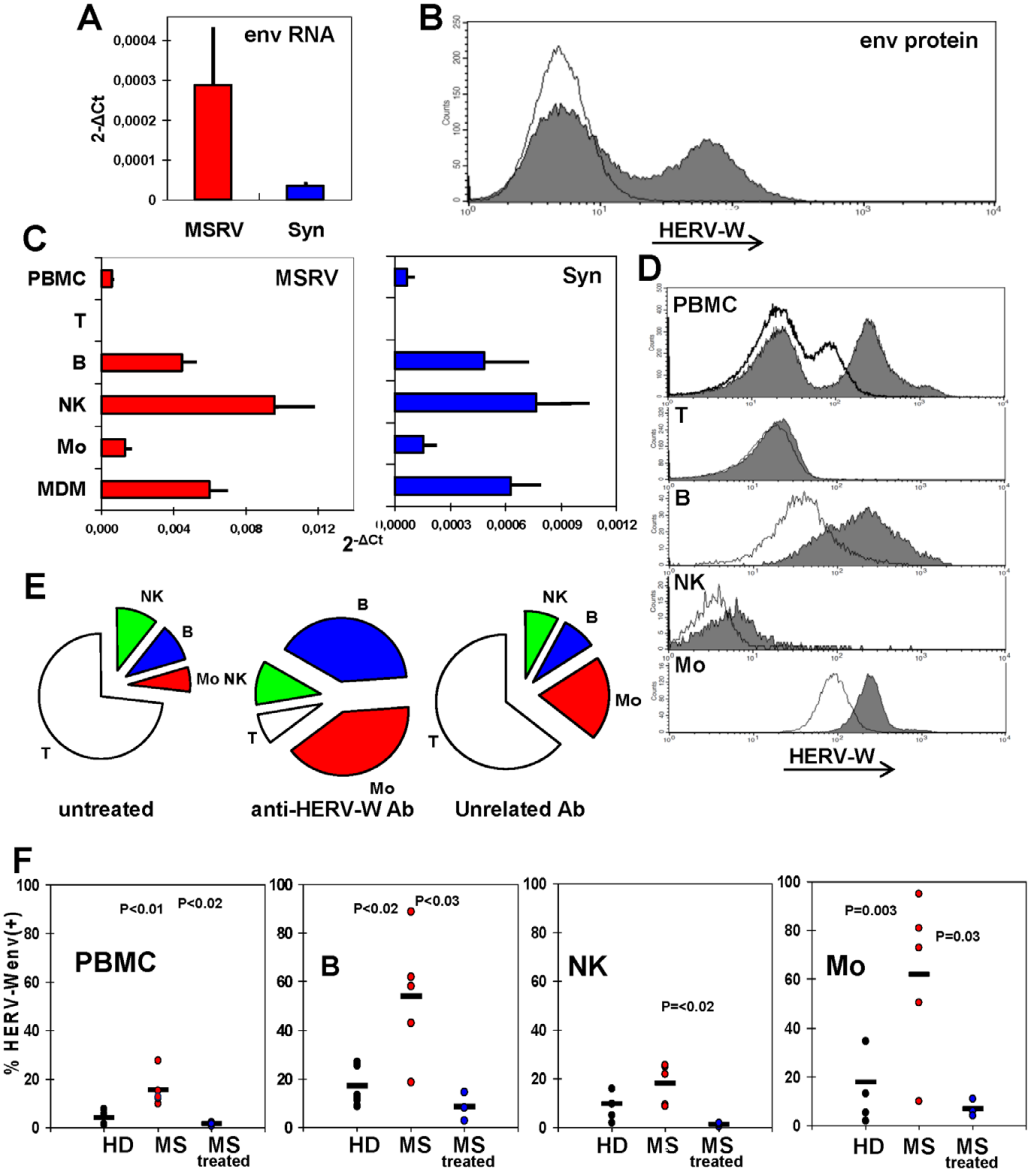
Basal expression of MSRVenv/ syncytin-1/HERV-Wenv in U87-MG astrocytes and PBMC subsets. A. Detection of MSRVenv and syncytin-1 mRNAs of U87-MG cells, by real time RT-PCR (means of three experiments run in duplicate, calculated by the 2^−ΔCt^ method; bars indicate standard deviation. **B**. Flow cytometry evaluation of the HERV-Wenv protein on U87-MG plasma membrane. Shaded histograms: HERV-Wenv-specific staining; open histograms: isotype control. **C.** MSRV*env* and Syncytin-1 mRNA expression on PBMC from MSRV(+) donors as such, and after immunobeads separation in CD3^+^T, CD19^+^ B, CD56^+^/CD19^−^/CD3^−^ NK and CD19^−^/CD3–/CD56^−^ monocyte subsets, and subsequent monocyte differentiation into MDM (bars indicate standard deviation). **D**. Flow cytometry evaluation of the HERV-Wenv protein on the membrane of PBMC from a representative MSRV(+) donor *in toto* and after sorting of the CD3^+^T, CD19^+^ B, CD56^+^/CD19^−^/CD3^−^ NK and CD19^−^/CD3^−^/CD56^−^ monocyte subsets. Shaded histograms: HERV-Wenv-specific staining; open histograms: isotype control. **E.** Cell populations distribution of PBMC from a representative MSRV(+) donor before, and after capture by magnetic beads charged with anti-HERV-Wenv or with an unrelated isotype antibody. The unprocessed PBMC and the cells retained by the immunobeads were sorted for T, B, NK and monocyte markers. The unretained cells were also analysed (not shown). **F**. Presence of surface HERV-Wenv protein in blood cells from five MSRV(+) HD, five untreated MS patients and three MS patients under effective therapy, evaluated by flow cytometry in PBMC *in toto* and after sorting of the CD3^+^T, CD19^+^ B, CD56^+^/CD19^−^/CD3^−^ NK and CD19^−^/CD3−/CD56^−^ monocyte subsets. Each dot represents an individual; horizontal bars represent the means. HERV-Wenv positivity of CD3^+^T cells was <2% for all samples (not shown).

As shown in the upper Panels of Figure 2, both MSRVenv and syncytin-1 mRNAs are detected in these cells, when reliable discriminatory real time RT-PCR assays are used (Figure 2A). When the cultures are in logarithmic phase of growth, more than one third of the cells expose the HERV-Wenv protein on the plasma membrane, as detected by flow cytometry (Figure 2B). Since no antibody capable of discriminate between MSRVenv and syncytin-1 is available, the immuno-staining could be due either to MSRVenv or syncytin-1, or both. Moreover, when comparing the expression of the env genes, it must be pointed out that the RT-PCR data detect the specific transcripts of the whole cells, while the flow cytometry data are referred to proteins present on the outer cell surface, only; thus they are representative only of a fraction of the total env protein products present in the cells (and are not indicative of the released products/virus particles).

The transcription of MSRV and syncytin-1 and the release of MSRV from PBMC in the blood and in culture has been shown repeatedly by several Authors, including us (reviewed in [5], [7]. To clarify whether MSRV and syncytin-1 are expressed preferentially by a particular cell subpopulation, PBMC from MSRV(+) HD were separated in T, B, NK and monocyte subsets, by immuno-specific adsorption to magnetic beads. Each subpopulation was divided in two aliquots, to be processed for real time RT-PCR and flow cytometry studies. Part of the monocytes was kept to differentiate in macrophages (MDM). As shown in Figure 2C, the mRNA amounts of MSRVenv and syncytin-1 of whole PBMC derive from an uneven accumulation in the various cell subsets. The T cells were totally negative, while high levels of transcripts were found in NK and B cells; in monocytes the expression was lower, but it increased by 3–4 folds with differentiation to MDM.

To monitor the presence of the HERV-Wenv protein on the plasma membrane, two different approaches were used. PBMC taken from MSRV(+) individuals were labelled with four different antibodies, each of them identified by a different fluorochrome. The flow cytometry of PBMC subsets is reported on Figure 2D. When the cells were sorted for HERV-Wenv-specific fluorescence only (upper histogram of Figure 2D), 34% of PBMC were shown to have HERV-Wenv on the plasma membrane. When the cells were sorted also for markers of PBMC subsets (lower histograms of Figure 2D), it appeared that the HERV-Wenv(–) PBMC fraction was mainly attributable to CD3^+^ T cells, according to the mRNA data of Figure 2C (and Figure 2E, see below); the sorting of CD19^+^ B cells, CD56^+^/CD19^−^/CD3^−^ NK cells, and CD19^−^/CD3−/CD56^−^ monocytes showed a significant shift of the peak of HERV-Wenv-specific fluorescence, compared to that of the unrelated antibody.

The other approach to monitor the distribution of HERV-Wenv(+) cells in PBMC is reported on Figure 2E: PBMC from MSRV(+) donors were mixed to magnetic beads charged either with anti-HERV-Wenv or with an unrelated isotype antibody. The unprocessed PBMC and the cells retained by the immunobeads were sorted for T, B, NK and monocyte markers. The unretained cells were also analysed (not shown). In the scanning of PBMC adsorbed to anti-HERV-Wenv-beads, a remarkable enrichment of the B and monocyte subsets was detected, that accounted for ∼80% of the HERV-Wenv-specific binding (∼5- and 6-fold enrichment of the B and monocyte subsets, respectively), while T cells dropped to ∼1/10^th^ of the percentage of whole PBMC, confirming the findings of Figure 2C and 2D. Sorting of cells retained through unspecific binding revealed a profile substantially similar to that of fresh PBMC, with a slight increase of the monocyte subset, likely to be due to the phagocytic properties of these cells.

Next, HERV-Wenv-specific immunostaining of PBMC from five untreated MS patients and three patients under therapy was compared to that of five MSRV(+) HD, with the same approach used for data of Figure 2D. When the cells were sorted for HERV-Wenv-specific fluorescence only, mean HERV-Wenv% immunostaining was 4.2±2.6, 15.5±7 and 1.5±0.8, for HD, untreated and treated MS patients, respectively. Similar data were obtained when the mean fluorescence intensity of the three groups was calculated (not shown). Of note the >10-fold reduction of HERV-Wenv positivity in the patients under therapy, a new finding, since the published reports were referred to inhibition of MSRV release in the plasma of IFN-treated patients only [19].

When the cells were sorted also for markers of PBMC subsets, the T cells were found almost HERV-Wenv(−) in all three groups (mean% immunostaining ≤1.5%, not shown). The values of HERV-Wenv-specific fluorescence of CD19^+^ B cells, CD56^+^/CD19^−^/CD3^−^ NK cells, and CD19^−^/CD3−/CD56^−^ monocytes are reported on Figure 2F. The untreated MS patients showed HERV-Wenv protein positivity higher than that of HD in all three cell subsets. Despite the low number of individuals, the statistical significance was reached for B cells and monocytes (P≤0.003). Notably, in the patients under therapy, HERV-Wenv positivity on the plasma membrane of all three subsets was highly reduced, and the differences with the untreated patients were statistically significant, in line with our proposal of MSRV inhibition as biomarker of MS therapy [19].

### EBV activates the expression of HERV-W/MSRVenv/syncytin-1 by astrocytes

**Figure 3:**
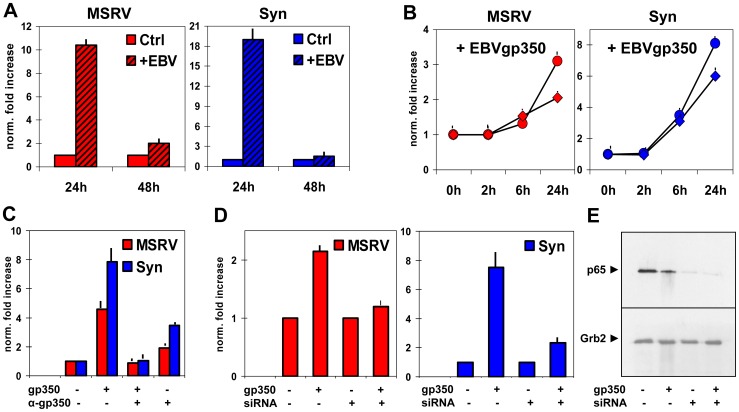
MSRVenv and syncytin-1 stimulation by EBV and EBVgp350 in U87-MG astrocytes. The levels of MSRVenv and syncytin-1 mRNAs were evaluated by real time RT-PCR as in Figure 2A, and are expressed as mean fold increases over controls (2^−ΔΔCt^ method). **A.** Expression of MSRV*env* and syncytin-1 in astrocytes cultured alone or co-cultured with the EBV-producer B95-8 cells for 24–48 h. **B.** Effects of 10 (•) or 100 (♦) ng/ml of EBVgp350 on MSRVenv and syncytin-1 mRNA accumulation during time. **C.** Suppression of EBVgp350 effects by its pre-incubation with anti-EBVgp350 neutralizing antibody. **D.** Requirement of NF-κB. Expression of MSRV*env* and syncytin-1 by U-87MG cells transfected or not with siRNA against the NF-κB p65 subunit and left overnight with/without 10 ng/ml of EBVgp350. E. Western blot assay of protein extracts from aliquots of the same cultures of Figure 3D. The relevant band was recognized by antibody against the NF-κB p65 subunit; the anti-Grb2 antibody was used to control the loading of equal amounts of proteins.

It has been reported that EBV can infect human astrocyte cell lines and primary foetal astrocytes [37]–[38]. By RT-PCR (not shown), we found that the U-87MG astrocytes cell line express the CD21 gene, whose protein is the main cell receptor used by EBV. Therefore, U-87MG cells were co-cultured with the EBV-producer B95-8 cell line, through Transwell membranes. Cell aliquots were harvested during time, to evaluate MSRVenv and syncytin-1 transcription by real time RT-PCR. Significant increases of MSRVenv and syncytin-1 transcription were detected at 24 h, that declined thereafter (Figure 3A). No MSRVenv/syncytin-1-specific amplification was detected in B95-8 cells, neither basal expression nor after co-culturing (not shown).

To verify that HERV-W activation in the co-cultures was specifically induced by EBV and not by factors released by the B95-8 cells, U-87MG cells were treated with graded amounts of EBVgp350, the major protein of EBV envelope and virus receptor. The Figure 3B reports the data of MSRVenv/syncytin-1-specific amplifications of a time-course experiment with 10–100 ng/ml of EBVgp350. As shown, the stimulation of MSRVenv and syncytin-1 occurs also with the EBVgp350 protein; for both elements the highest effect was obtained with 10 ng/ml of EBVgp350 and 24 h of treatment. The HERV-Wenv activation is specifically due to EBVgp350, since it was abolished by pre-treatment of EBVgp350 with anti-EBVgp350 antibody (Figure 3C), but not by an unrelated one (not shown). A slight effect was observed with the anti-EBVgp350 antibody alone; it might be due to cross-reactivity due to similarity of epitopes on the plasma membrane of the astrocytes, or to contaminants of the antibody preparation.

It is known that the binding of EBVgp350 to CD21 induces the activation of the NF-κB transcription factor [39]. To verify if NF-κB plays a role on MSRVenv and syncytin-1 activation, we silenced NF-κB by transient transfection of U87-MG cells with silencer RNA (siRNA) specific for the NF-κB p65 subunit; then the cells were treated with EBVgp350, and analysed by real time RT-PCR (Figure 3D) and Western blot (Figure 3E). In conditions where the NF-κB p65 subunit was silenced, as proven by the absence of the specific band in Western blot, the stimulation of the two retroelements by EBVgp350 was dramatically reduced. This finding is in line with the report of a role for NF-κB in the regulation of the syncytin-1 promoter by TNFα, and IFNγ [40], through a NF-κB-responsive element located within the enhancer (cellular) domain of the promoter. The Figure 3 shows that syncytin-1 is twice more responsive than MSRVenv to EBV. Since the promoters of the two elements have identical viral moieties [40], the difference should lie in the upstream regulatory cellular region of the two promoters.

### Modulation of MSRVenv and syncytin-1 by EBVgp350 in PBMC

**Figure 4:**
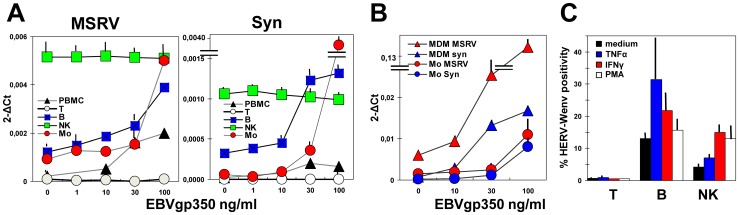
Expression of MSRVenv and syncytin-1 by PBMC subsets exposed to EBVgp350 or proinflammatory cytokines. **A.** Levels of MSRV*env* and syncytin-1 mRNAs of PBMC from MSRV(+) HD treated overnight with 1–100 ng/ml of EBVgp350, either as such or separated in T, B, NK and monocyte subsets. **B.** Comparison of the MSRVenv and syncytin-1 mRNA levels of monocytes and MDM after overnight EBVgp350 treatment. A and B: Data are the means of three experiments run in duplicate, calculated by the 2^−ΔΔCt^ method; **C.** Expression of the HERV-Wenv protein on the plasma membrane, evaluated by flow cytometry as present env-specific positivity of PBMC treated for 24 h with TNFα (1 ng/ml), IFNγ (1000 IU/ml), or PMA (50 NM); the bars indicate standard deviations.

The most probable site of EBV/HERV-W interactions should be the circulating cells. Thus PBMC from MSRV(+) HD, either as such or separated in T, B, NK and monocyte subsets, of the same type of experiments whose basal MSRVenv and syncytin-1 transcription is shown in Figure 2C, were treated overnight with graded amounts of EBVgp350; the Figure 4A reports the data of MSRVenv/syncytin-1 mRNA accumulation. Part of the monocytes was left to differentiate in MDM, before treatment with EBVgp350, and the data are reported in Figure 4B. As shown, the amounts of both MSRVenv and syncytin-1 in whole PBMC cells exposed to EBVgp350 were increased dose-dependently (up to ∼10-fold for both genes, at the highest dose), and derive from uneven mRNA levels in the various cell subsets. The T cells, that are HERV-Wenv(−), were not induced by EBV to a *de novo* transcription, nor effects were observed on NK cells, despite high basal levels of expression for both genes. The B and monocyte subsets, instead, were stimulated by EBVgp350. Of interest the EBV effect on the monocytes, whose basal levels of transcripts were lower than those of B cells, but that were more sensitive to EBVgp350, reaching values of the same range of those of treated B cells (MSRVenv), or higher (syncytin-1). The effect of EBVgp350 on syncytin-1 was higher than that on MSRVenv, suggesting that also in blood cells the two promoters are regulated differently.

Monocyte differentiation into MDM increased by 3–4 folds MSRVenv and syncytin-1 transcription (as shown also in Figure 2C), and amplified the effects of EBVgp350. The Figure 4B shows that MDM exposed to 100 ng/ml of EBVgp350 expressed mean levels of MSRVenv and syncytin-1 78 and 84 folds those of monocytes, respectively. The finding that the highest effect of EBV on HERV-W expression was seen on MDM might be relevant for the possible contribute of HERV-W elements to MS pathogenesis, since it has been reported that inflamed cells of the blood-brain-barrier promote the differentiation and/or maturation of migrating monocytes, and that this could shape auto-aggressive immune processes occurring early in CNS inflammation [41]. Furthermore, since monocytes may constitute up to 8% of circulating cells and can infiltrate inflamed tissues, including the brain, this cell subset seems to be responsible for the bulk of HERV-W expression, during EBV infection. On the other hand, any episode that induces the production of proinflammatory cytokines, as it occurs during MS exacerbations, will increase both monocyte migration to the brain, their differentiation into macrophages, and expression of potentially pathogenic HERV-Wenv [50], [69].

The lack of effect of EBVgp350 on T and NK cells could be due to the absence of receptors for EBV on these cells. To verify if other stimuli can modulate HERV-W expression in NK and T cells, PBMC were exposed to either TNFα or IFNγ proinflammatory cytokines, or to phorbol-12-myristate-13-acetate (PMA). As shown on Figure 4C, NK and B cells were stimulated similarly to hyperexpress HERV-Wenv proteins, while no *de novo* HERV-Wenv expression was observed in T cells. Thus HERV-W elements can be activated also in NK cells, when the proper stimuli are given; since also PMA is active, this suggests an involvement of the protein kinase C signalling enzyme.

## Discussion

Late EBV infection/seroconversion has been shown to be a risk factor for the development of MS in the next 5–20 years [2], with a maximum of a ∼ ten-fold increase in MS risk for DRB1*15-positive persons with a history of IM [22]–[26]. The mechanism by which the late EBV infection is linked to MS risk is still unknown; so far, no convincing data support unequivocally a direct etiologic role of EBV or prove its preferential presence, expression or reactivation in MS patients with respect to controls [2], [29]. It is highly debated whether EBV infects MS brain and contributes directly to CNS immunopathology [42]–[43]. Some hypotheses have been proposed as to how EBV infection could potentially promote autoimmunity and nervous tissue damage in MS: primary EBV infection might facilitate activation of auto-reactive lymphocytes, through recognition of pathogen-derived molecules by Toll-like receptors and other pattern recognition receptors [40]. A report by Serafini *et al*. [43] described rare brain structures similar to ectopic B-cell follicles, identified as major sites of EBV persistence, in autopsy samples of 9/13 patients with secondary progressive MS (e.g. in the late stages of the disease, 12–35 years from MS onset, thus presumably decades since primary EBV infection). Based on this, a direct action of EBV within the brain was postulated, with autoreactive and polyspecific T cells that should be maintained in the presence of continuous antigen exposure, due to EBV persistence [40]. Other studies could not confirm the presence of ectopic B-cell follicles, and reported that CNS EBV infection is rare in MS brain, indicating that EBV infection is unlikely to contribute directly to multiple sclerosis brain pathology in the vast majority of cases [42]. The report of a recent workshop dedicated to EBV in the brain, signed also by the group of Serafini [44], agreed that the presence of EBV-infected cells in MS brains remains highly controversial and that unequivocal proof of EBV infection in MS lesions is still lacking.

On the other hand, an association between MS and either one or both the two potentially neuropathogenic MSRV and syncytin-1 HERV-W elements has been repeatedly and independently reported by several groups, including us. Extracellular, coat-protected, MSRV RNA sequences have been found increased in the blood and spinal fluids of MS patients of several cohorts of Caucasians [12], [14], [15], [45]–[49]. Several studies reported increased expression of Syncytin-1 in MS patients [11], [12], [34], [50]–[51]. MSRV presence was associated to the conversion from optic neuritis to full-blown MS [20]; MRSV presence/load strikingly paralleled MS temporal and clinical stages [15] and was related to worse prognosis in the next ten years [16]–[18]. Notably, MSRV viremia became undetectable in MS patients under therapy with interferon*β* [19], and was proposed as prognostic biomarker of the disease and of therapy outcome.

Since the expression of potentially neuropathogenic MSRV/syncytin-1 HERV-W elements parallels the behaviour of the MS disease, and the role of EBV in MS induction is still unknown, a link between EBV and HERV-W elements seemed possible. This also in view of the fact that some herpesviruses can activate the expression of retroelements: the herpes simplex-1 virus was shown to transactivate HERV-W/MSRV elements [52]–[55], and the expression of HERV-K18 is activated by EBV [56]–[59] and by human herpesvirus-6 [60]–[61]. To evaluate the possible interactions between EBV and HERV-W/MSRV/syncytin-1, we focused on cells related to MS pathogenesis and\or target of EBV infection: (i) the astrocytes, that help demyelination, by promoting inflammation, damage of oligodendrocytes and axons, and glial scarring, but also allow remyelination by acting on oligodendrocyte proliferation, and differentiation [62], and (ii) PBMC subsets, that are the main site of EBV replication and persistence, and play pivotal roles in MS in immune defence and immunopathogenesis.

The *in silico* survey of the human genome reported in the present paper shows that full length HERV-W*env* DNA sequences are present on ten chromosomes, and that three additional ones have elements containing ≥80% of the HERV-W*env* gene (Figure 1). As for transcripts, data from GenBank indicate that only the ERVW-1 locus on chromosome 7q21.22 is transcribed in a full length *env* mRNA, that can be translated in a complete protein, the syncytin-1. All the other HERV-W*env* genes present in the current version of the human genome, either are not transcribed, or originate HERV-Wenv fragments with stop codons and other gene alterations [63]–[65].

The majority of HERV-W*env* studies used tools not discriminating the components of the HERV-W family [35]. At the RNA level, MSRV*env* and syncytin-1 share >94% identities [12]; at the protein level, no antibody specific for a unique HERV-W are available so far [5]. Therefore the origin of the HERV-Wenv, whose expression has been found associated to MS is still debated. It has been suggested that MSRV might be either an exogenous HERV-W, or a nonubiquitous replication-competent member, or a partly defective but nonubiquitous copy, seldom complemented or recombined within the HERV-W family [4]–[5], [66]–[70]. So far, the real nature of MSRV remains to be elucidated. Whatever the origin of MSRV is, a meta-analysis showed that ∼9% of Caucasian blood donors have circulating virionic MSRV/HERV-W RNA [5].

The real-time RT-PCR assays employed in the present study amplify selectively either MSRV*env* or syncytin-1 sequences [34]. As shown on Table 1, the latter primers recognize only the transcripts from the ERVW-1 element on the chromosome 7q21.22, while MSRV*env*-specific primers, apart from the extracellular MSRV*env* gene, can amplify RNAs from the element on chromosome Xq22.3 only; this element can originate an incomplete protein, that is unglycosylated, does not form oligomers, and is located intracellularly [64]; thus the present version of the human genome (that presumably does not contain sequences derived from MS patients) does not contain complete HERV-W*env* elements, recognized by the MSRV*env*-specific primers, that can originate a full length MSRVenv protein. Therefore the HERV-Wenv protein(s) on the plasma membrane detected by the anti-HERV-Wenv antibody employed in the present study could derive from the element present on the chromosome 7q21.22 and/or from MSRV, only.

Firstly, we monitored the expression of MSRV*env* and syncytin-1 in astrocytes and PBMC, cells involved in MS pathogenesis, and/or that could host the interactions among the above viruses. As reported on Figure 2 A and B, in U87-MG astrocytes a basal expression of both HERV-W*env* genes was detected at the mRNA level; accordingly, the HERV-Wenv protein was detected on the plasma membrane. This could be expected, since HERV-Wenv expression by the astrocytes of active lesions in the brain of MS patients has been reported [11], [12], [71].

The PBMC from both MS patients and healthy MSRV(+) donors express MSRV*env* and syncytin-1 mRNAs, and have the HERV-Wenv protein on the outer surface of the plasma membrane (Figure 2 C–F). The two elements, however, are not uniformly expressed by all the PBMC, but derive from an uneven expression in the various cell subsets. As shown in Figure 2E, when PBMC were adsorbed to anti-HERV-Wenv-beads, ∼80% of the cells retained by the beads are B cells and monocytes. High levels of expression of HERV-W/MSRVenv/syncytin-1 were found in NK and B cells; in monocytes the expression was lower, but it increased with differentiation to MDM. This is a novel finding for endogenous retroviruses, but was shown for exogenous retroviruses, such as HIV, whose expression in chronically infected monocytes increases with differentiation to MDM [72]. The expression of HERV-W in NK cells has not been reported previously. These cells appear to express the highest levels of both MSRVenv and syncytin-1 MSRVenv (see Figure 2C and basal levels of Figure 4A). How HERV-W activation could influence the dual role played by NK cells in MS, as inflammatory mediators of damage and as immune regulatory cells suppressing harmful inflammatory activity [73], remains to be elucidated. The T cells were found totally negative for HERV-Wenv expression, both as HERV-Wenv protein on the outer cell surface and as intracellular MSRV*env* and Syncytin-1 transcripts. This finding indicates that the absence of the HERV-Wenv protein on the membrane of T cells (Figures 2 and 4), that was reported by flow cytometry also by Brudek *et al*. [51], is due to absence of *env* transcripts in the T cells, that cannot be induced by none of the stimuli active on other cell subsets (Figure 4).

Comparison of the HERV-Wenv expression of blood cells from HD and MS patients, either naive or under effective therapy (Figure 2F) showed that in the patients with active MS there is an increase of 3–4-fold in the percentage of cells expressing the HERV-Wenv protein on the membrane, for both whole PBMC and B, NK, and monocyte subsets, indicating that HERV-Wenv activation is similar in all the already HERV-W-positive PBMC subsets, while the T cells remained negative. The specific immunostaining sharply decreased in the patients under therapy. This is a new finding, since our published report of inhibition of MSRV release during effective anti-MS therapy was referred to patients treated with interferonβ [19]; in that case, the inhibition of HERV-W/MSRV release could be explained in terms of antiviral effects (related or not to the anti-MS action of interferon, that was postulated to be due to its modulation of T and B cell activity and effects on the blood-brain barrier). In the case of the patients of Figure 1F, treated with glatiramer acetate, the anti-HERV-W effect could be related to its induction of anti-inflammatory cytokines [74], while that of azathioprine that interferes with DNA synthesis could be related to its inhibition of proliferation of cells, especially of T and B cells. In any case, our findings confirm that efficacious anti-MS therapy is paralleled by inhibition of HERV-W expression.

Following infection by EBV, U87-MG astrocytes increased the transcription of both MSRV*env* and syncytin-1 (Figure 3A). There is no need of EBV entry or expression, since the effect was seen also after exposure to the EBVgp350 major envelope protein. The effect is abolished by silencing NF-κB (Figure 3C–D), a transcription factor that is implicated in the production, by activated astrocytes, of pro-inflammatory cytokines: this, in turn, induces further release of cytokines, that contribute *in vivo* to exacerbating the neurodegenerative process [75], and that *in vitro* stimulate HERV-W*env* expression in astrocytes (unpublished data) and PBMC (Figure 4C, and [69]. The HERV-Wenv proteins use, as their cell surface receptors, the sodium-dependent neutral amino acid transporter type 1 and 2 (ASCT-1 and ASCT-2), involved in the cellular intake of amino acids [76]. Binding of HERV-Wenv to ASCT-1 or -2 receptors might create critical reduction in the amino acid intake necessary for their normal activity. Of note, reduced detection of these ASCT receptors at the neuronal surface in schizophrenic cortex was reported [77] and connected with receptor modulation or masking by HERV-Wenv protein [6].

In the PBMC exposed to EBVgp350, MSRV*env* and syncytin-1 are stimulated in B cells and monocytes, but not in T cells, nor in the highly expressing NK cells. The latter cells, but not the T cells, are activated by proinflammatory cytokines. Thus HERV-W elements can be stimulated also in NK cells, when the proper stimuli are given. Of particular interest, in our opinion, is the monocyte/macrophage (M/M) cell compartment. These cells are the most responsive to EBVgp350, reaching env levels higher than those detected in B cells, particularly after cell differentiation into macrophages. Since the monocytes can easily pass across the endothelia, and through the blood-brain barrier, the HERV-W expression (and release) by monocyte-macrophages can reasonably account for the bulk of expression (and effects) of these elements. One must remind the fundamental roles played by M/M, not only in the clearance of virions or virally infected cells by phagocytosis, in antigen processing, leading to the activation of the humoral immune response and to generation of cytotoxic T-lymphocytes, but also activation of the innate immunity, release of soluble factors/cytokines, NK activation. Notably, neurotoxic mediators released from M/M are thought to be involved also in the pathogenesis of neuroAIDS, and accumulation of activated M/M in the brain correlates with neurological injury: monocytes infected with HIV have enhanced migratory capacity through the blood–brain barrier; within the CNS, they differentiate and mediate neuronal injury and dysfunction through the secretion of viral proteins, inflammatory factors, excess glutamate, and other neurotoxins [78]–[79]. Increased BBB permeability is a characteristic hallmark of the CNS alterations observed in MS, suggesting a causal relationship between inflammatory cell recruitment into the CNS and the blood–brain barrier dysfunction [80], and HERV-Wenv production by activated M/M could contribute to the pathogenicity. To be noted, in this respect, that the HERV-Wenv protein has been shown to activate innate immunity through CD14/TLR4 and to promote Th1-like responses [81].

## Conclusions

The present study demonstrates that basal expression of MSRVenv and syncytin-1 is present in U87-MG astrocytes and in PBMC from MS patients and some healthy donors; among PBMC subsets, MSRV*env* and syncytin-1 are expressed by NK, B cells and monocytes, but not by T cells. It is shown for the first time that EBV infection or cell exposure to the EBVgp350 protein stimulates the expression of MSRVenv and syncytin-1, up to the final protein product on the plasma membrane, and that this activation involves the NF-κB pathway. Novel findings are also HERV-Wenv expression by NK cells, and differential expression and modulation of the HERV-Wenv protein in PBMC subsets from MS patients (untreated and under therapy) and MSRV-positive healthy donors. It is demonstrated that *in vitro* interactions among the two proposed MS-cofactors, HERV-W and EBV, occur in cells from blood and from brain. It is likely that it might occur also *in vivo*. Therefore, in an individual, the possibility of the activation of the immunopathogenic and neuropathogenic potential of HERV-W by superinfecting EBV is concrete, both in blood and in brain. The original sin could lie in abnormal host reactions to a late primary EBV infection, that often leads to IM; of note, a mathematical model addressing the age-dependence of IM, suggests that variation in host antibody responses and the complexity of the pre-existing cross-reactive T-cell repertoire, both of which depend on age, may play important roles in the aetiology of IM [82]. During IM, an abnormal activation of circulating cells occurs. Among other effects, EBV proteins might deregulate also the expression of HERV-Ws, in B and M/M cells. Activation of HERV-W elements- could induce an immune cascade activated by HERV-Wenv protein; activated cells, especially the M/M cells, could also go across the blood-brain barrier. In the blood, the EBV/HERV-W interactions might induce immunopathogenic phenomena linked to HERV-Wenv superantigenic properties. In the brain, the EBV/HERV-W interaction could establish also toxic mechanisms against the oligodendrocytes, thus inducing inflammation, demyelination and axonal damage. Local stimulation of astrocytes, by proinflammatory cytokines and other factors might activate further expression of HERV-Ws, contributing to the neuropathogenity, since presence of MSRV in spinal fluids of MS patients is an early event, predictor di worse progression [16]–[18].

Our findings reinforce the hypothesis of a possible involvement of these viruses in MS pathogenesis, with the possibility for MSRV of a direct role of effector of pathogenicity, and for EBV of an initial trigger of future MS, years later, since the possibility of the activation of the immuno- and neuro-pathogenic potential of HERV-W by superinfecting EBV is concrete, both in blood and in brain.

During the revision of the paper, a multicentric study has been published [89] on the association of MSRVenv antigenemia with MS, that independently confirmed our findings on increased positivity/expression levels of PBMC MSRV RNA and of MSRV DNA copy number in MS patients, and expression of MSRVenv protein within brain MS lesions; the HERV-W Env protein detection in MS sera confirmed our data on the association of circulating HERV-W/MSRVenv with MS.

## Materials and Methods

### Ethics Statement

The study was approved by the Sassari AOU Ethics Committee. The patients and the volunteers gave written informed consent.

### Cells

U-87MG: human glioblastoma-astrocytoma, epithelial-like cells, grown in Dulbecco's modified Eagle's medium 10% foetal calf serum (FCS, Life Technologies, Inc., Carlsbad, CA). B95-8: marmoset B lymphoblastoid cells, chronically infected and producing EBV. PBMC were isolated from heparinized peripheral blood of MSRV(+) donors (HD, and MS patients) by layering on Ficoll/Hipaque [19]. The PBMC and the B95-8 were maintained in RPMI-1640 medium 10% FCS (Invitrogen, San Giuliano Milanese, IT). Cell viability was assessed with the Trypan blue exclusion method.

### Blood samples

HD: Five already studied MSRV(+) volunteers of our cohort [34], three females, two males (mean age 43±15.0 years). MS patients: eight patients (six females, two males; mean age 39±13.0 years) attending the Department of Neurosciences, University of Sassari, with a diagnosis of relapsing remitting MS according to international guidelines [83]. Three patients were untreated and three were under (effective) therapy (one was treated with 2.6 mg/kg/day of azathioprine and two were treated with 20 mg/day of glatiramer acetate). Table 2 reports the anamnestic, clinical and therapy data of the patients.

**Table 2:** Clinical and therapy features of MS patients.

Patient	Age	Sex	MS years	MS phase	EDSS	Treatment	Response
MSu1	26	F	<1	RR	≤3	none	n.a.*
MSu2	59	F	<1	RR	≤3	none	n.a.
MSu3	19	F	<1	RR	≤3	none	n.a.
MSu4	60	M	32	RR	6	none	n.a.
MSu5	30	M	3	RR	≤3	none	n.a.
MSt1	32	F	5	RR	≤3	azathioprine	yes
MSt2	45	F	2	RR	≤3	glatiramer acetate	yes
MSt3	22	F	1	RR	≤3	glatiramer acetate	yes

* : n.a.: not applicable.

### Separation of PBMC subpopulations

The PBMCs were fractionated using the Magnetic Cell Sorting kits (MACS, Miltenyi Biotec, Auburn, CA): NK cells were obtained by negative selection (NK Cell Isolation Kit, Miltenyi Biotec); to obtain B, T and monocytes, PBMC were treated with anti-CD4/anti-CD8 (for T cells) and anti-CD14 monoclonal antibody (for monocytes), respectively (BD Biosciences Becton Dickinson, San Jose, CA) and mixed with microbeads (anti-mouse Ig microbeads, MACS, Miltenyi Biotec), according to the manufacturer's protocols (>95% purity was achieved). The same method was used to select from PBMC the HERV-Wenv(+) cells, using a polyclonal rabbit anti-HERV-Wenv (Allele Biotec, San Diego, CA) and the anti-rabbit Ig microbeads (MACS, Miltenyi Biotec). MDM were obtained by incubating monocytes for 6 days in RPMI 20% FCS.

### Co-culture studies

The U87-MG cells were plated on Transwell® Receiver plates (Costar® Snapwell membranes, Corning Inc., Lowell, MA); exponentially growing B95-8 cells were added on the Transwell® Reservoir plates 0.4 µm pore size. After incubation for 1–2 days, cells were collected and processed for mRNA studies.

### Treatment with EBVgp350

Semi-confluent U-87MG astrocytes, PBMC and PBMC subpopulations were treated overnight with 10–100 ng/ml of recombinant EBVgp350 protein, then were harvested and processed for further studies. Fully purified EBVgp350 was kindly provided by A.J. Morgan, University of Bristol, UK, and its endotoxin content was <25 pg/ml. [84].

### EBVgp350 neutralization assay

The cells were treated for 24 h either with 10 ng/ml of EBVgp350 (protein as such, or pre-incubated for 2 h at room temperature with anti-EBVgp350 antibody (Santa Cruz Biotechnology, Inc., Santa Cruz, CA) or with anti-mouse IgG (Abcam, Cambridge, UK) as an unrelated antibody, or with medium. The cells were then harvested and processed for quantification of the transcripts of interest, as below.

### Treatment with cytokines and PMA

The PBMC were exposed overnight to either human recombinant TNFα, IFNγ, or PMA, at the doses of 1 ng/ml, 1000 IU/ml, and 50 nM, respectively (Sigma-Aldrich, St. Louis, MO), then were harvested and processed for RT-PCR and flow cytometry.

### RNA extraction and Real-time PCR

RNAs were extracted from 50.000 cells, by mRNA Dynabeads® kit (Dynal Biotech, Oslo, NO) and retrotranscribed; selective amplification of MSRVenv and syncytin-1 sequences was obtained by utilizing the corresponding primer pairs, as published in [34]. The comparison of the relative amounts of the two sequences in RNAs was carried out by the 2^−ΔCt^ and 2^−ΔΔCt^ Methods, based on the different amplification of the gene of interest with respect to the glyceraldehyde-3-phosphate dehydrogenase housekeeping gene [85].

### siRNA interference

Transient knockdown of the NF-κB p65 subunit was performed with p65-specific siRNA. using the transfection reagents of Santa Cruz Biotechnology as published [40]. Briefly, 24 h after seeding, semiconfluent U87-MG cells were rinsed once with Optimem (Invitrogen, Carlsbad, CA); transfection was performed with 50 pmols of siRNA, at the final concentration of 0,125 μg/μl (Santa Cruz Biotechnology, Inc.) by the method of Surabhi and Gaynor [86] in foetal serum-free medium for transfection (Santa Cruz Biotechnology, Inc.). After 6 h was added 1 ml of medium with double concentration of foetal calf serum and antibiotics. After 12 h, 10 ng/ml of EBVgp350 were added, and cultures incubated overnight, then collected and processed for mRNA and Western blot studies.

### Western Blot analysis

The expression of the NF-κB p65 subunit was detected in cell lysates by immunoblotting using monoclonal antibodies against the NF-κB p65 subunit (Santa Cruz Biotechnology, Inc.) at a 1∶1000 dilution. The growth factor receptor-binding protein 2 (Grb2) was included as a loading control, and detected using a mouse anti-Grb2 antibody (Sigma Aldrich, St. Louis, MO) at a dilution of 1∶15,000. Secondary antibodies used were HRP-conjugated anti-mouse IgG at a 1∶10,000 (Santa Cruz Biotechnology Inc.), according to [40].

### Flow cytometry

The detection of HERV-Wenv protein on the plasma membrane was performed using anti-HERV-Wenv rabbit polyclonal antibody (Allele Biotec) and secondary fluorescein isothiocyanate-conjugated goat anti-rabbit IgG (Sigma-Aldrich). The isotype control was a pre-immune rabbit serum (Santa Cruz Biotechnology, Inc). To determine the phenotype of cell subpopulations, PBMC were stained with allophycocyanin-conjugated anti-CD3, phycoerythrin-conjugated anti-CD19, and peridinin-chlorophyll-conjugated anti-CD56 antibodies (BD Biosciences). As isotype control, an anti-rabbit IgG antibody was used (Santa Cruz Biotechnology, Inc). The analysis was performed as detailed elsewhere [87]. Cells were analysed on a FACS Calibur flow cytometer using Cells Quest software (Becton-Dickinson). The area of positivity was determined using an isotype-matched antibody; a total of at least 5×104 events for each sample were acquired.

### HERV-Wenv mining

The National Centre for Biotechnology Information (NCBI) BLASTN and BLASTX programs were used [36] for mining HERV-Wenv sequences from the GenBank human genome database, using the following queries: MSRV*env* sequence (PV14 MSRV clone, GenBank accession number AF331500), Syncytin-1 (ERVEW1*env* coding sequence, accession number NM_014590.3), MSRV*env*-specific 166 bp and syncytin-1-specific 100 bp amplicons generated with the corresponding discriminatory primers described in [34].

### Statistical analyses

The significance of the results was evaluated by means of the Epi Info™ 7 database and statistics software program, (CDC/WHO, Atlanta, GA, USA). P values of less than 0.05 were determined to be significant.

## Supporting Information

Table S1 Chromosomal location of MSRVenv-type and Syncytin-1-type HERV-Wenv genes.Table S1a: HERV-Wenv genes in human DNA, (>80 of total env, as detected by the PV14 MSRVenv sequence (AF331500). In silico nucleotide BLAST search [36]in the current version of the human genome, using as query the MSRVenv sequence (PV14 MSRV clone, GenBank accession number AF331500). From the initial BLAST-identified regions, the sequences covering ≥80% of the genes were selected. (DOC). Table S1b: HERV-Wenv genes in human DNA, (>80 of total env, as detected by the ERVEW1 coding sequence (NM_014590.3). In silico nucleotide BLAST search as for Table S1a, using as query the syncytin-1 sequence (ERVEW1env coding sequence, GenBank accession number NM_014590.3).
(DOC)
