# Supplementary material for: Expression and Activation by Epstein Barr Virus of Human Endogenous Retroviruses-W in Blood Cells and Astrocytes: Inference for Multiple Sclerosis
Source: PLoS One. 2012 Sep 27;7(9):e44991. doi: 10.1371/journal.pone.0044991 (PMC3459916; doi:10.1371/journal.pone.0044991)
Supplement: Table S1 — Chromosomal location of MSRVenv-type and Syncytin-1-type HERV-Wenv genes.Table S1a: HERV-Wenv genes in human DNA, (>80 of total env, as detected by the PV14 MSRVenv sequence (AF331500). In silico nucleotide BLAST search [36]in the current version of the human genome, using as query the MSRVenv sequence (PV14 MSRV clone, GenBank accession number AF331500). From the initial BLAST-identified regions, the sequences covering ≥80% of the genes were selected. (DOC). Table S1b: HERV-Wenv genes in human DNA, (>80 of total env, as detected by the ERVEW1 coding sequence (NM_014590.3). In silico nucleotide BLAST search as for Table S1a, using as query the syncytin-1 sequence (ERVEW1env coding sequence, GenBank accession number NM_014590.3). (DOC) [file pone.0044991.s001.doc]

**Supporting information file**

**Table S1: Chromosomal location of MSRV*env*-type and Syncytin-1-type HERV-W*env* genes.**

**Table S1a: HERV-W*env* genes in human DNA* (>80 of total *env*, as detected by the PV14**

**MSRV*env* sequence (AF331500)**

| **Chromosome** | **Length** | **Identities** | **(%)** | **Gaps** | **Accession Number** |
| --- | --- | --- | --- | --- | --- |
| **X** | 1630 | 1599/1630 | (98) | 2/1630 | NT_011651.17, NW_927717.1 |
|  | 1596 | 1466/1596 | (91) | 22/1596 | NT_167197.1, NW_927700.1 |
|  | 1559 | 1435/1559 | (92) | 22/1559 | NW_001842359.1 |
|  | 1439 | 1429/1439 | (99) | 0/1439 | NW_001842391.1 |
|  |  |  |  |  |  |
| **3** | 1629 | 1509/1629 | (93) | 14/1629 | NT_005612.16 |
|  | 1629 | 1506/1629 | (92) | 14/1629 | NW_001838880.2, NW_921807.1 |
|  | 1366 | 1273/1366 | (93) | 8/1366 | NW_001838884.2 |
|  |  |  |  |  |  |
| **4** | 1636 | 1507/1636 | (92) | 16/1636 | NW_922162.1 |
|  | 1636 | 1505/1636 | (91) | 16/1636 | NT_022778.16, NW_001838914.1 |
|  | 1633 | 1500/1633 | (91) | 13/1633 | NW_001838920.1 |
|  | 1633 | 1499/1633 | (91) | 13/1633 | NT_016354.19, NW_922217.1 |
|  | 1634 | 1496/1634 | (91) | 25/1634 | NW_001838921.1 |
|  |  |  |  |  |  |
| **5** | 1631 | 1520/1631 | (93) | 14/1631 | NT_006713.15, NW_001838934.1 |
|  | 1631 | 1519/1631 | (93) | 14/1631 | NW_922607.1 |
|  | 1630 | 1516/1630 | (93) | 6/1630 | NT_034772.6 |
|  | 1630 | 1515/1630 | (92) | 6/1630 | NW_001838952.2 |
|  | 1630 | 1500/1630 | (92) | 8/1630 | NW_922751.1 |
|  |  |  |  |  |  |
| **7** | 1632 | 1521/1632 | (93) | 18/1632 | NT_007933.15,NW_001839064.2,  NW_923574.1, NT_079595.2 |
|  |  |  |  |  |  |
| **9** | 1393 | 1289/1393 | (92) | 7/1393 | NW_924539.1 |
|  | 1393 | 1288/1393 | (93) | 7/1393 | NT_008470.19 |
|  | 1393 | 1288/1393 | (92) | 7/1393 | NW_001839236.2 |
|  |  |  |  |  |  |
| **11** | 1435 | 1344/1435 | (93) | 10/1435 | NT_009237.18 |
|  | 1435 | 1341/1435 | (93) | 10/1435 | NW_001838022.2, NW_925006.1 |
|  |  |  |  |  |  |
| **12** | 1634 | 1508/1634 | (92) | 9/1634 | NT_029419.12, NW_001838057.1, NW_925351.1 |
|  |  |  |  |  |  |
| **14** | 1633 | 1525/1633 | (93) | 7/1633 | NT_026437.12,NW_001838111.1, NW_925539.1 |
|  |  |  |  |  |  |
| **15** | 1631 | 1507/1631 | (92) | 14/1631 | NT_010194.17,NW_001838218.2, NW_925884.1 |
|  |  |  |  |  |  |
| **17** | 1650 | 1510/1650 | (91) | 41/1650 | NW_926817.1 |
|  | 1650 | 1509/1650 | (91) | 41/1650 | NT_010783.15,NW_001838434.2 |
|  |  |  |  |  |  |
| **18** | 1634 | 1525/1634 | (93) | 15/1634 | NW_003315956.1 |
|  |  |  |  |  |  |
| **20** | 1462 | 1363/1462 | (93) | 7/1462 | NW_001838666.1, NW_927339.1 |
|  | 1463 | 1362/1463 | (93) | 9/1463 | NT_011362.10 |

***:** Four additional HERV-Wenv sequences containing ≥80 of the gene are found in GenBank (accession numbers GX389322, GZ041480, EA780163, GX389295), without chromosomal location; these sequences share the extra stop codon at position 115, and have >98% identities with the HERV-Wenv sequences of chromosome X. Thus, it is likely that they are located on chromosome X.

**Table S1b: HERV-W*env* genes in human DNA (>80 of total *env*, as detected by the ERVEW1 coding sequence (NM_014590.3).**

| **Chromosome** | **Length** | **Identities** | **(%)** | **Gaps** | **Accession Number** |
| --- | --- | --- | --- | --- | --- |
| **X** | 1632 | 1526/1632 | (93) | 18/1632 | NT_011651.17 |
|  | 1596 | 1459/1596 | (91) | 33/1596 | NT_167197.1 |
|  | 1559 | 1424/1559 | (91) | 33/1559 | NW_001842359.1 |
|  | 1442 | 1366/1442 | (95) | 6/1442 | NW_001842391.1 |
|  |  |  |  |  |  |
| **3** | 1632 | 1501/1632 | (92) | 32/1632 | NT_005612.16 |
|  | 1632 | 1498/1632 | (92) | 32/1632 | NW_001838880.2 |
|  | 1365 | 1273/1365 | (93) | 16/1365 | NW_001838884.2 |
|  |  |  |  |  |  |
| **4** | 1631 | 1490/1631 | (91) | 21/1631 | NW_001838920.1 |
|  | 1631 | 1489/1631 | (91) | 21/1631 | NT_016354.19 |
|  | 1636 | 1489/1636 | (91) | 41/1636 | NW_001838921.1 |
|  | 1635 | 1489/1635 | (91) | 26/1635 | NT_022778.16, NW_001838914.1 |
|  |  |  |  |  |  |
| **5** | 1633 | 1516/1633 | (93) | 30/1633 | NT_006713.15, |
|  | 1633 | 1514/1633 | (93) | 30/1633 | NW_001838934.1 |
|  | 1633 | 1513/1633 | (93) | 24/1633 | NT_034772.6 |
|  | 1633 | 1512/1633 | (93) | 24/1633 | NW_001838952.2 |
|  |  |  |  |  |  |
| **7** | 1617 | 1617/1617 | (100) | 0/1617 | NT_007933.15, NW_001839064.2, NT_079595.2 |
|  |  |  |  |  |  |
| **9** | 1393 | 1290/1393 | (93) | 19/1393 | NT_008470.19 NW_001839236.2 |
|  |  |  |  |  |  |
| **11** | 1433 | 1331/1433 | (93) | 18/1433 | NT_009237.18 |
|  | 1433 | 1330/1433 | (93) | 18/1433 | NW_001838022.2 |
|  |  |  |  |  |  |
| **12** | 1634 | 1500/1634 | (92) | 21/1634 | NT_029419.12 |
|  |  |  |  |  |  |
| **14** | 1634 | 1517/1634 | (93) | 21/1634 | NT_026437.12 NW_001838111.1 |
|  |  |  |  |  |  |
| **15** | 1633 | 1502/1633 | (92) | 30/1633 | NT_010194.17, NW_001838218.2 |
|  |  |  |  |  |  |
| **17** | 1650 | 1515/1650 | (92) | 53/1650 | NT _010783.15 NW_001838434.2 |
|  |  |  |  |  |  |
| **18** | 1633 | 1521/1633 | (93) | 25/1633 | NW_003315956.1 |
|  |  |  |  |  |  |
| **20** | 1461 | 1358/1461 | (93) | 17/1461 | NT_011362.10 |
|  | 1461 | 1356/1461 | (93) | 17/1461 | NW_001838666.1 |
